# Control within a virtual environment is correlated to functional outcomes when using a physical prosthesis

**DOI:** 10.1186/s12984-018-0402-y

**Published:** 2018-09-05

**Authors:** Levi Hargrove, Laura Miller, Kristi Turner, Todd Kuiken

**Affiliations:** 1 Shirley Ryan AbilityLab, 355 E. Erie Street, Chicago, IL 60611 USA; 2 Departments of Physical Medicine and Rehabilitation and Biomedical Engineering, Northwestern University, 663 Clark St, Evanston, IL 60208 USA

**Keywords:** Prosthetics, Myoelectric control, Pattern recognition, Outcomes

## Abstract

**Background:**

Advances such as targeted muscle reinnervation and pattern recognition control may provide improved control of upper limb myoelectric prostheses, but evaluating user function remains challenging. Virtual environments are cost-effective and immersive tools that are increasingly used to provide practice and evaluate prosthesis control, but the relationship between virtual and physical outcomes—i.e., whether practice in a virtual environment translates to improved physical performance—is not understood.

**Methods:**

Nine people with transhumeral amputations who previously had targeted muscle reinnervation surgery were fitted with a myoelectric prosthesis comprising a commercially available elbow, wrist, terminal device, and pattern recognition control system. Virtual and physical outcome measures were obtained before and after a 6-week home trial of the prosthesis.

**Results:**

After the home trial, subjects showed statistically significant improvements (*p* < 0.05) in offline classification error, the virtual Target Achievement Control test, and the physical Southampton Hand Assessment Procedure and Box and Blocks Test. A trend toward improvement was also observed in the physical Clothespin Relocation task and Jebsen-Taylor test; however, these changes were not statistically significant. The median completion time in the virtual test correlated strongly and significantly with the Southampton Hand Assessment Procedure (*p* = 0.05, *R* = − 0.86), Box and Blocks Test (*p* = 0.007, *R* = − 0.82), Jebsen-Taylor Test (*p* = 0.003, *R* = 0.87), and the Assessment of Capacity for Myoelectric Control (*p* = 0.005,*R* = − 0.85). The classification error performance only had a significant correlation with the Clothespin Relocation Test (*p* = 0.018, *R* = .76).

**Conclusions:**

In-home practice with a pattern recognition-controlled prosthesis improves functional control, as measured by both virtual and physical outcome measures. However, virtual measures need to be validated and standardized to ensure reliability in a clinical or research setting.

**Trial registration:**

This is a registered clinical trial: NCT03097978.

## Background

Major upper-limb amputation affected over 40,000 Americans as of 2005 [1], with over 11,000 additional wrist disarticulation or higher-level amputations between 2005 and 2013 [2]. The impairment of bimanual dexterity caused by amputation interferes with basic activities of daily living; routine activities such as driving, household work, and leisure activities; and limits employment opportunities. Currently, the most effective treatment is use of a prosthesis, and recent advances in prosthetic technology, including myoelectric devices with multi-articulating hands, pattern recognition–based control systems, and surgical techniques such as targeted muscle reinnervation (TMR) [3] have been developed to improve prosthetic function. However, a functional performance remains challenging.

As prosthetic limbs and control systems become more advanced and costly it is important to quantify performance benefits that these technology improvements provide to users. Outcome measures may also be useful to help track progress through rehabilitation protocols and indicate specific areas which required additional therapy. The Academy of Prosthetics and Orthotics Upper Limb Prosthetics Outcome Measure committee provided recommendations for measuring functional effectiveness of prosthetic treatment/occupational therapy [4]. Rather than relying on a single test across all patients and phases of device development, they recommended using multiple test formats to capture all aspects of performance. Promising tests included the Assessment for Capacity of Myoelectric Control (ACMC), the Southampton Hand Assessment Procedure (SHAP), a modified Box and Blocks test, the Jebsen-Taylor Test, and a Clothespin Relocation task. Of these tests, only the ACMC has been validated and demonstrated to have good test-retest reliability for the field of upper-limb prosthetics [5]. The remaining tests have been identified as promising tests to use, particularly when performing research and development studies in the field of upper-limb prosthetics [6].

An alternative method of assessing performance is to use virtual environments or serious gaming—video games or virtual environments designed for training purposes. Proponents of these tools promote their economic benefits, their manageable and rapid development, and the availability of powerful computing and processing technologies as factors driving the recent success and popularity of simulated environments for clinical and research applications. Virtual tools have been developed for stroke rehabilitation [7], assessment of children with cerebral palsy [8], and for other neuromuscular disorders [9]. Several virtual environments have been proposed for myoelectric control applications.

Virtual environments for myoelectric control have evolved from rudimentary graphical user interfaces to more life-like virtual avatars and real-time practice environments, with performance tasks such as virtual clothespin movement tasks [10], posture matching tasks [11], or Fitts-law style target acquisition tasks. Alternative approaches have abstracted the experience away from controlling a prosthetic limb, instead using myoelectric signals as inputs to engage in commercially available video games, such as Guitar Hero™ [12] or custom-designed serious games [13].

Intuitively, one would expect improved performance or testing scores within a virtual environment to translate into better prosthesis control, which would in turn lead to better functional outcomes. However, this assumption has not been thoroughly tested. Powell et al. [14] showed that upper limb prosthesis users could control a virtual prosthesis better after practicing pattern recognition control in a virtual environment across multiple days; however, functional tests with a physical prosthesis were not reported. Recently van Dijk et al. [13] demonstrated transfer of myoelectric control skills after serious gaming, but only if the game was designed to encourage behaviors specific to controlling a prosthesis. In addition, van Dijk’s study was limited in that it was performed with able-bodied subjects.

The primary objective of this study was to determine the relationship between performance on a virtual test—the Target Achievement Control (TAC) test—and performance with a physical prosthesis. The secondary objective was to determine whether, after extensive occupational therapy, allowing subjects to practice with a pattern recognition–controlled prosthetic arm during a 6-week home trial would further improve functional outcomes.

## Methods

Nine individuals with transhumeral level amputations who had previously undergone targeted muscle reinnervation (TMR) surgery were recruited for the study (Table 1). All subjects, with the exception of P9, were myoelectric prosthesis users prior to enrolling into the study but not all routinely used their prosthesis.

**Table 1:** Patient demographics

Patient	Age (years)	Time since amputation (years)	Time since TMR (years)	Side	Gender	Etiology	Terminal device
P1	35	4	3	R	M	Trauma (military)	Hook-ETD
P2	45	2	1	R	M	Trauma (train)	Hand
P3	54	6	< 1	L	M	Trauma (military)	Hook-ETD
P4	58	5	1	L	M	Sarcoma	Hook-ETD
P5	25	6	6	L	M	Trauma	Hook-ETD
P6	31	8	7	L	M	Trauma (military)	Hook-Greifer
P7	27	2	1	R	M	Trauma (crushing)	Hook-Greifer
P8	31	1	1	R	M	Trauma (MVA)	Hook-ETD
P9	44	1	< 1	R	F	Trauma (MVA)	Hand

*PGT* Prosthesis Guided Training

TMR has previously been described in detail [3]. In this surgical procedure, severed motor nerves that previously controlled arm and hand function, are transferred onto denervated *target* muscles—muscles that no longer serve a biomechanical function after amputation. After reinnervation, target muscles serve as biological amplifiers of the motor control commands intended for the missing arm and thus provide physiologically appropriate EMG control signals, making prosthesis control intuitive. For example, after reinnervation of a segment of biceps muscle by the transferred median nerve, contraction of that muscle—as the user attempts to close their missing hand—generates EMG signals that close the motorized hand, conversely, reinnervation of a segment of triceps by the transferred distal radial nerve generates EMG signals that control hand open.

**Fig. 1:**
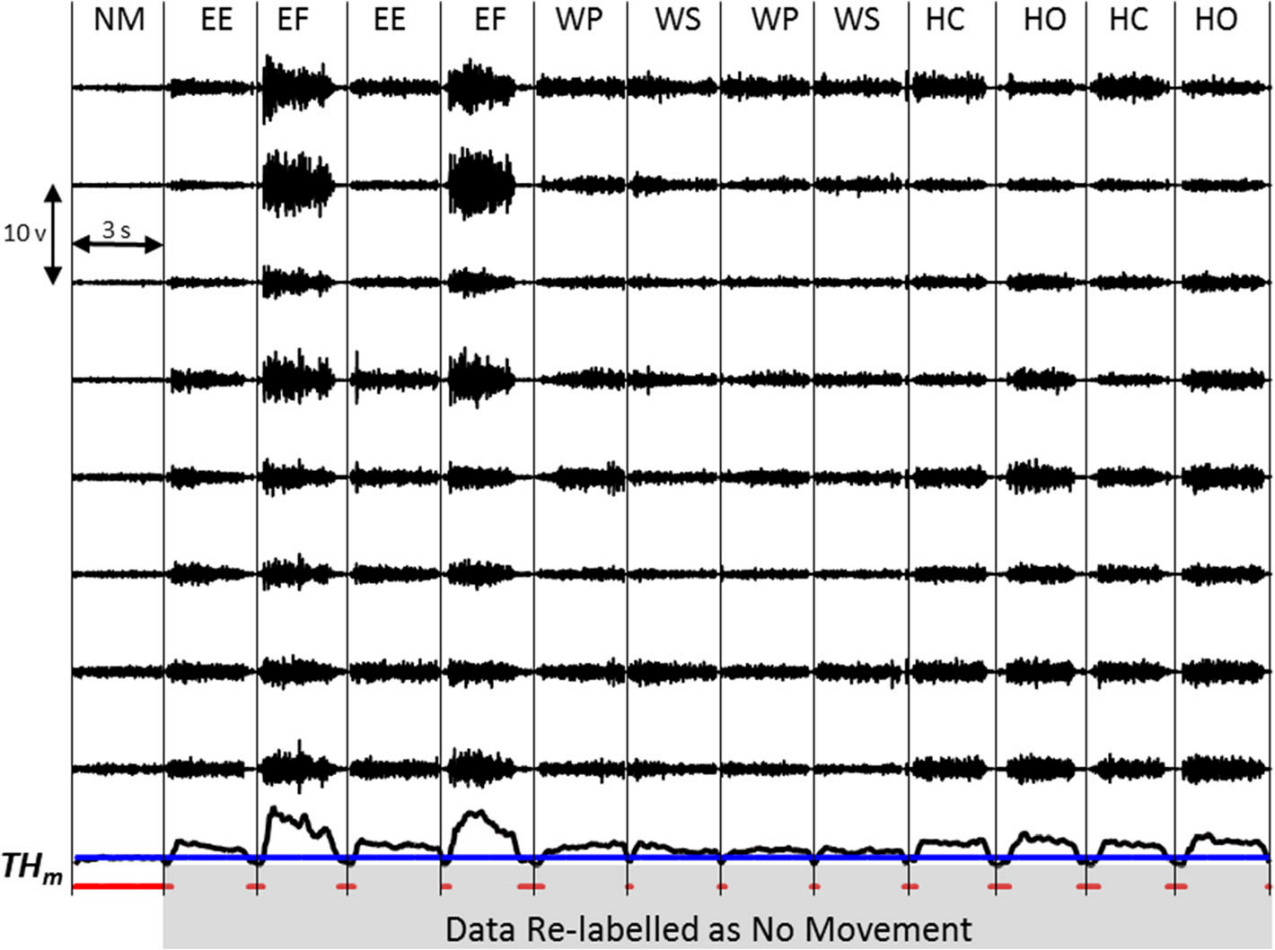
Representative data from a prosthesis-guided training sequence. Data labels are provided by prosthesis movement; the resulting EMG patterns are used to train a pattern recognition system as described by Kuiken et al. [16]

A custom-fabricated prosthesis was created for each subject using a Boston Digital Elbow (Liberating Technologies Inc.), a Wrist Rotator (Motion Control Inc.), and a single degree-of-freedom (DOF) terminal device of their choice (Table 1). The prosthesis was capable of the following powered movements: elbow flexion (EF), elbow extension (EE), wrist pronation (WP), wrist supination (WS), terminal device open (TDO), terminal device close (TDC), in addition to no movement (NM). All subjects, except P9, were fit with custom-fabricated thermoplastic elastomeric gel liners (Alps Inc.). P9 was fit with a custom rolled silicone liner to minimize length of the prosthesis. Stainless steel electrodes were embedded into the wall of the liners, and EMG signals were transmitted, through stretchable conductive fabric leads, to the electronics at the distal end of the liner. A grid of electrodes was used, as described in previous work [15], rather than placing electrodes over specific muscles. Briefly, signals were amplified and digitized using a Texas Instruments ADS1299 chip, sampled at 1000 Hz, and transmitted to an embedded controller. The pattern recognition algorithm, described in detail in Kuiken et al. [16], interpreted the signals and sent appropriate commands to the prosthesis. Amplifier gains were set on a subject-specific basis, with a typical value of 2000, and data were digitally filtered between 70 and 450 Hz. A recalibration switch was laminated into the outer wall of each socket so that the users could recalibrate the pattern recognition system, using prosthesis-guided training [16], whenever they desired. An example of the EMG signal patterns collected during a representative recalibration sequence are shown in Fig. 1.

Seven of the nine subjects were naïve to pattern recognition control. While their prosthesis was being constructed, an occupational therapist taught these subjects the concepts of pattern recognition and instructed them how to make repeatable and distinct muscle contractions [17]. During this initial training phase, subjects were given visualization exercises to strengthen their muscles, which has been shown to improve users’ ability to make repeatable and distinct contractions for pattern recognition control [18], but received no real-time control feedback. Once all subjects could perform consistent contractions for each intended prosthesis movement, EMG data from four repetitions of each prosthesis movement, held for 3 s, were collected used to train the pattern recognition algorithm. A series of images presented on a computer monitor were used to guide subjects through the data collection procedure. Immediately after data collection, subjects performed three blocks of the TAC Test [11]. The user was required to move a flesh-colored virtual limb to match the posture of a translucent grey-colored virtual target limb in real time, within a 15-s time frame, essentially a timeout ceiling that limited the length of the trials. Each block comprised a set of 12 postures. Target limb postures were selected such that the subject had to control each DOF of the prosthesis through 75% of its range of movement, stop within the posture location (±5° of each DOF), and maintain the target posture location for 2 s. Outcome metrics for this virtual outcomes test included (i) the number of postures successfully acquired within their allotted 15-s time frames, and (ii) the median completion time required to match the set of postures in a block. Median completion time was used rather than the mean, as the data were skewed by the 15-s timeout ceiling. Immediately after completion of the three blocks, data from four repetitions of each movement, held for 3 s, were collected and used to evaluate the classification error rate of the pattern recognition system.

Subjects were then fit with the physical prosthesis and received occupational therapy over 3–4 consecutive days for approximately 6 h per day. Subjects then performed a set of outcome measures (pre-home trial testing) that included the Southampton Hand Assessment Protocol (SHAP), the Jebsen-Taylor Test of Hand Function, three repetitions of the Box and Blocks test, and three repetitions of the Clothespin Relocation task. These measures were chosen to evaluate hand, wrist, and elbow function and were activities that could be reasonably completed with a physical prosthesis.

Individuals then took the device home for a minimum of 42 days (6 weeks) of home use. If the prosthesis needed to be repaired, or if the user had a valid, documented reason for not wearing a myoelectric prosthesis—e.g., an extreme sports competitions, a beach vacation, being sunburned—then additional time was added to ensure at least 6 weeks of usage. The controller logged the amount of time that the prosthesis was powered on and the number of times the patient recalibrated the control system. After completion of the home trial, subjects returned to the laboratory and repeated the virtual and physical outcome measures described above (post-trial testing), in addition to completing the ACMC.

For the SHAP and the classification error rate measures, where only a single pre- and post-trial score was available, a one-tailed paired = t-test was used to compare differences. For other outcome measures where multiple trials were recorded, a repeated measures ANOVA with the subject as random factor, and pre/post trial condition and trial number were fixed factors. A correlation analysis using the Pearson coefficient was used to determine relationships between virtual and physical outcome measures.

## Results

All subjects wore the device at home, and could successfully recalibrate the device (Table 2). Subject P2 typically removed the prosthesis while it was still powered on, and thus it was not possible to accurately determine wear-time for this subject. The occasional recalibration failures observed were primarily due to broken wires.

**Table 2:** Prosthesis usage during home trial

Patient	Number of successful/attempted PGT sessions	Total number of days worn	Total wear time (hrs)
P1	7/7	9	45
P2	39/39	18	–
P3	73/77	41	181
P4	56/57	58	365
P5	10/10	36	88
P6	20/20	14	28
P7	18/18	20	127
P8	38/38	28	69
P9	60/60	32	88

Designates statistical significance at the *p* < 0.05 level. *CRT* Clothespin Relocation Test. *PGT* Prosthesis Guided Training

Classification error rates are frequently reported to characterize the performance of upper-limb pattern recognition control systems. Pre-trial classification error and TAC test data were available from six of the nine subjects: two subjects had prior experience controlling the virtual prosthesis so they did not complete pre-home trial virtual environment testing, and data from one subject was lost due to a computer malfunction. Post-trial data were available from all nine subjects.

**Fig. 2:**
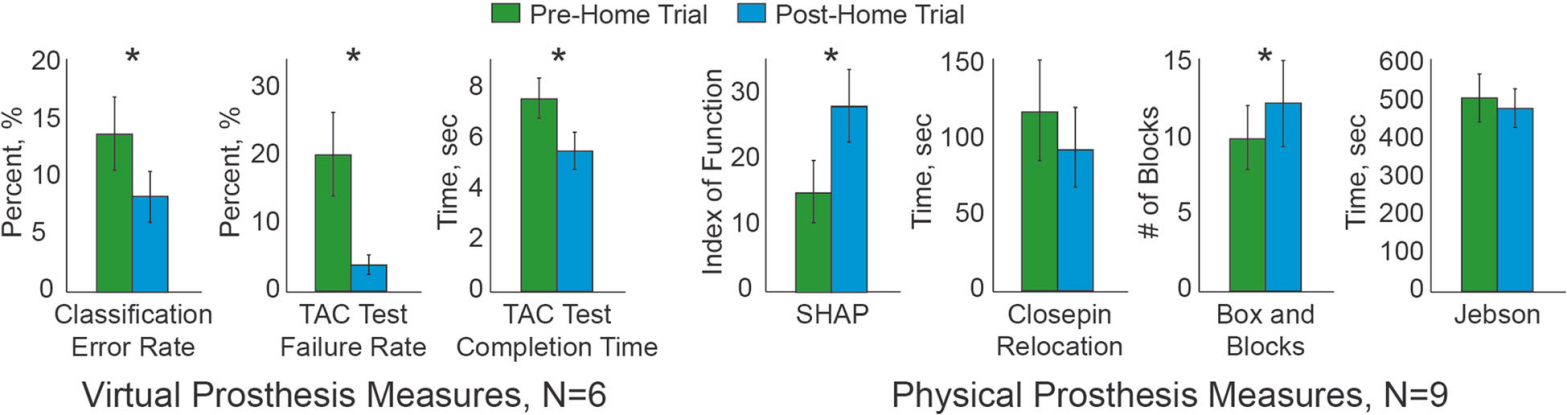
Outcome measures when using a virtual prosthesis (left) or a physical prosthesis (right). Measures were performed before and after a 6-week home trial. *Denotes statistical significance at *p* = 0.05

After the home trial, average classification error across subjects dropped from 13.4 to 8.3%, which was significantly lower (*p* = 0.03) (Fig. 2). All TAC test performance metrics also improved significantly after the home trial: failure rate improved from 19.9 to 3.7% to Y (*p* = 0.001), and completion time dropped from 7.5 to 5.5 s (*p* = 0.007).

All nine subjects completed the outcome measures using the physical prosthesis. All outcome measures for use of the physical prosthesis tended to improve after the home trial; however, only the SHAP (*p* = 0.001) and the Blocks and Box test (*p* = 0.03) showed statistically significant improvements.

**Fig. 3:**
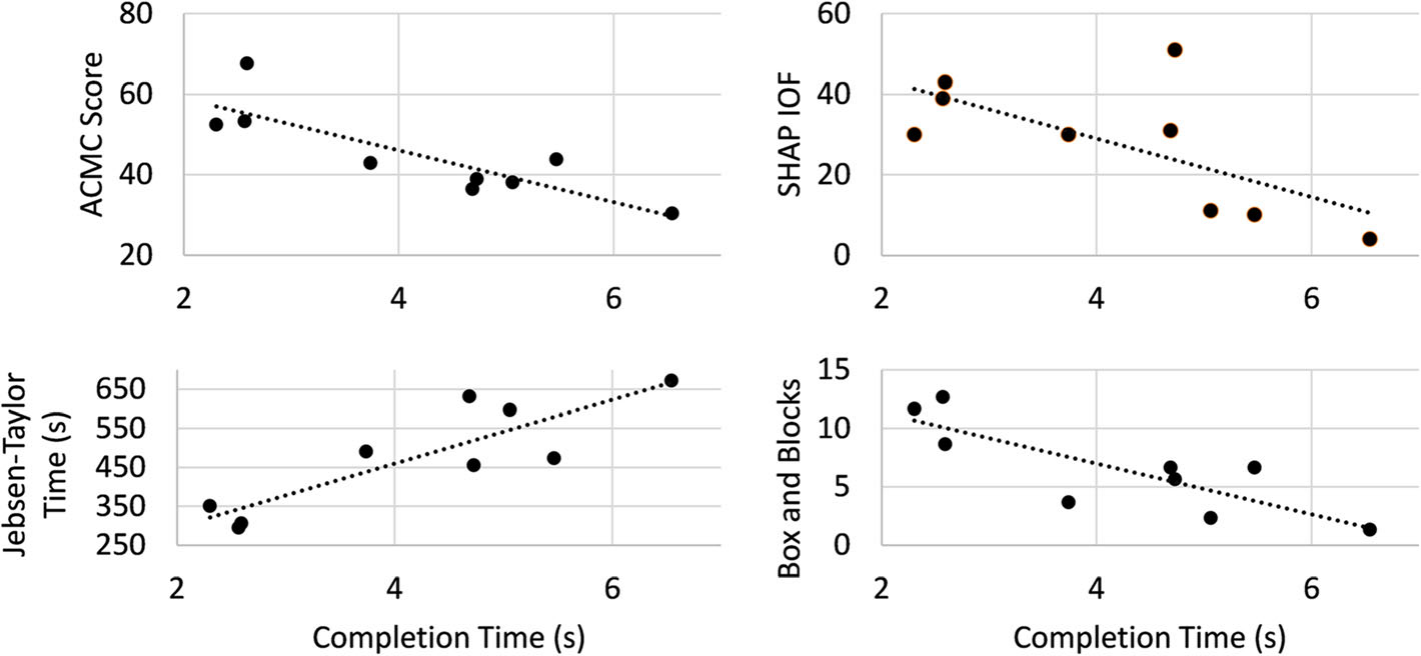
Statistically significant relationships between virtual and physical outcome measures. Each relationship was strong, with a Pearson correlation coefficient |R| > 0.75

We performed Pearson correlation analyses to investigate the relationship between TAC test outcome measures and physical outcome measures (Table 3). We found strong and statistically significant correlations between TAC completion times and several of the physical outcome measures for the post home-trial outcomes (Fig. 3). Correlations between TAC completion time and the SHAP, Box and Blocks test, and ACMC were negative, i.e., faster completion times were associated higher test scores, indicating better performance in these physical measures. The correlation between completion time and the Jebsen-Taylor test was positive, i.e., faster completion times were associated with faster test times, indicating better physical task performance. We also found that the classification error rate, which is an offline measure of performance, did not show statistically significant correlations with any virtual measure, but did correlate strongly with performance in the Clothespin Relocation task (*p* = 0.018).

**Table 3:** Pearson correlation coefficients, R, between virtual and physical outcome measures

Predictor	SHAP	CRT	Box and blocks	Jebsen-Taylor	ACMC
Completion Time	−0.86*	0.30	−0.82*	0.87*	−0.85*
Failure Rate	0.19	0.52	− 0.54	0.27	− 0.37
Classification Error	−0.13	0.76*	−0.46	0.39	−0.31

*Denote statistical significance at the *p* = 0.05 level

## Discussion

Pattern recognition–based myoelectric control systems have seldom been systematically evaluated outside of a controlled laboratory environment. Most studies are performed to evaluate control algorithm classification error rates, or to evaluate ways to make classification error rates more robust to environmental factors, prolonged use, or non-ideal conditions [18–20]. However, initial clinical case series evaluating commercially available pattern recognition systems have reported positive patient experiences [21]. Our results show that users are capable of using pattern recognition–controlled myoelectric limbs within their home environment. Although the amount of time the prosthesis was worn and the frequency with which the control system was recalibrated was variable, the results of outcome tests using the pattern recognition–controlled prosthesis were equivalent or superior to measures recorded after subjects used the same prosthesis during an equivalent home trial, using conventional control [22].

We observed statistically significant improvements in the SHAP and Blocks and Box test after a 6-week home trial. The Clothespin Relocation task and the Jebsen-Taylor test also showed trends toward improvement that were not statistically significant. We also found statistically significant improvements in classification error rate and all outcome metrics associated with the virtual TAC test after the trial. Clearly, subjects learned to control the device better during the home trial.

Limited published data has supported the hypothesis that patients learn to perform more consistent, distinct contractions over time with practice, leading to speculation that this would lead to improved control of a pattern recognition–controlled prosthesis [14, 18, 23]. Our data support this idea, as classification error rates improved significantly (*p* < 0.05) after the home trial. However, classification error rate had a strong, statistically significant correlation with the Clothespin Relocation task, but did not correlate significantly with any of the other physical outcome measures with any TAC outcome metric. In contrast, TAC test completion time correlated strongly and significantly with all physical outcome measures except the Clothespin Relocation task. These results reinforce the growing body of literature supporting the importance of performing online testing, preferably with individuals with amputations, rather than relying solely on classification error analyses during offline experiments to evaluate control [10, 24–26].

This work is important because it demonstrates a correlation between virtual test measures and physical performance. However, the study has several limitations. As with any correlation analysis, correlation does not imply causation. Without further study, we cannot say that working within a virtual environment will transfer to better functional outcomes, although the work of van Dijk et al. suggests that this may be true under certain situations [13]. Furthermore, developing validated and reliable outcome measures in the field of upper-limb prosthetics is a challenging problem. As for physical outcome measures, virtual measures must be thoroughly described to ensure consistent administration, analysis, and interpretation. For example, the ACMC has an established test-retest, inter-rater, and intra-rater reliability and clinical interpretation guidelines. The TAC test may be made easier or more difficult by changing the length of time allowed to acquire postures, the number of DOFs needed to acquire the posture, the distance the virtual limb must be moved, and the tolerances required for matching the target posture. Finally, the results of this study are only applicable to transhumeral amputees. Future investigations will need to be performed to develop appropriate relationships between virtual and physical outcome measures for individuals with other levels of upper limb amputation.

## Conclusions

Providing users with an opportunity to use a pattern recognition–controlled prostheses in their home-environment for at least 6 weeks resulted in improved functional control, as measured by a set of outcome measures. This highlights the need for practice, in addition to comprehensive occupational therapy, before assessing outcomes. Improvements were seen in both the offline performance metric of classification error rate and in real time control outcome measures when controlling a virtual or physical prosthesis. Finally, we found that some outcome measures, particularly the TAC test completion time, correlated strongly with physical outcome measures. Future work will further investigate these relationships using a more standardized test configuration, and with a broader population of subjects.

## Abbreviations

ACMC: Assessment for capacity of myoelectric control
ANOVA: Analysis of variance
CRT: Clothespin relocation task
DOF: Degrees of freedom
EE: Elbow extension
EF: Elbow flexion
HC: Hand close
HO: Hand open
NM: No movement
PGT: Prosthesis-guided training
SHAP: Southampton Hand Assessment Procedure
TAC: Target acquisition control
TMR: Targeted muscle reinnervation
WP: Wrist pronation
WS: Wrist supination
